# Comment on “Soft Mappings Space”

**DOI:** 10.1155/2019/6903809

**Published:** 2019-01-10

**Authors:** T. M. Al-shami

**Affiliations:** Department of Mathematics, Sana'a University, Sana'a, Yemen

One of the celebrated findings obtained in general topology reports that every compact subset of a Hausdorff space is closed. In this investigation, we demonstrate that this finding need not be true via soft topology. In 2014, Ozturk and Bayramov [1] discussed some properties of a soft Hausdorff space which defined in [2, 3] and its relationships with a soft compactness notion. However, they made an error, as we observe, in [Theorem 34, p.p.5] which investigated a relationship between soft closed set and soft compact Hausdorff space. To illustrate this mistake, we provide a counterexample and then we conclude under what conditions this result can be generalized via soft topology.

We draw the attention of the readers to the fact that there are different kinds of soft Hausdorff spaces introduced in the literature. Some of them depend on the distinct ordinary points (see, for example, [2–4]) and the others depend on the distinct soft points (see, for example, [5–7]).

First of all, we recall the following three definitions which will be needed throughout this manuscript.

**Definition 1 (see [2, 3]).**

A soft topological space (*X*, *τ*, *A*) is said to be a soft *T*_2_-space (or soft Hausdorff) if for every *x* ≠ *y* ∈ *X*, there are two disjoint soft open sets (*G*, *A*) and (*F*, *A*) such that *x* ∈ (*G*, *A*) and *y* ∈ (*F*, *A*).

**Definition 2 (see [4]).**

For a soft set (*G*, *A*) over *X* and *x* ∈ *X*, we say that *x*⋐(*G*, *A*) if *x* ∈ *G*(*a*), for some *a* ∈ *A*; and we say that x ⋐ (G,A) if *x* ∉ *G*(*a*), for each *a* ∈ *A*.

**Definition 3 (see [4]).**

A soft set (*G*, *A*) over *X* is said to be stable if there exists a subset *U* of *X* such that *G*(*a*) = *U*, for each *a* ∈ *A*.

Now, we mention the alleged results [Theorem 34, p.p.5] as originally proposed in [1].

**Theorem 4 .**

Every soft compact subset (*F*, *A*) of a soft Hausdorff space (*X*, *τ*, *A*) is soft closed.

In what follows, we construct an example to show that the above theorem is not valid in general.

**Example 5 .**

Let *A* = {*a*_1_, *a*_2_} be a set of parameters and let the set of real numbers *ℛ* be the universe set. Then we show that a collection *τ* = {(*G*, *A*): either 1 ∈ (*G*, *A*) such that (*G*^*c*^, *A*) is finite or 1 ⋐ (G,A)} is a soft topology on *ℛ* as follows:

- Since $1\in \tilde{ℛ}$ and $(\tilde{ℛ}{)}^{c}=\tilde{\Phi }$ is finite, then $\tilde{ℛ}\in \tau$ and since 1 ⋐ Φ~, then $\tilde{\Phi }\in \tau$.
- Let (*G*_*i*_, *A*) ∈ *τ*. Then we have the following three cases for arbitrary union $\tilde{⋃}(G_{i},A)$ and for finite intersection $\tilde{⋂}(G_{i},A)$:
  1. 1 ∈ (*G*_*i*_, *A*), for each *i*, then $1\in \tilde{⋃}(G_{i},A)$ and $1\in \tilde{⋂}(G_{i},A)$. So $\tilde{⋃}(G_{i},A)\in \tau$ and $\tilde{⋂}(G_{i},A)\in \tau$.
  2. 1 ⋐ (Gi,A), for each *i*, then 1 ⋐ ⋃~(Gi,A) and 1 ⋐ ⋂~(Gi,A). So $\tilde{⋃}(G_{i},A)\in \tau$ and $\tilde{⋂}(G_{i},A)\in \tau$.
  3. 1 ∈ (*G*_*i*_, *A*), for some *i*, then $1\in \tilde{⋃}(G_{i},A)$ and [$1\in \tilde{⋂}(G_{i},A)$ or 1 ⋐ ⋂~(Gi,A)]. So $\tilde{⋃}(G_{i},A)\in \tau$ and $\tilde{⋂}(G_{i},A)\in \tau$.
- Thus *τ* is closed under arbitrary soft union and finite soft intersection.

Hence (*ℛ*, *τ*, *A*) is a soft topology. Moreover, it is a soft Hausdorff space. On the other hand, a soft set (*F*, *A*), which defined as *F*(*a*_1_) = {1} and *F*(*a*_2_) = {5}, is a soft compact subset of $\tilde{ℛ}$. But it is not soft closed.

**Remark 6 .**

It should be noted that the given soft topological space above is considered as a version of Fort space via soft topology.

Before we investigate the correct form of [Theorem 34, p.p.5] in [1], we present the next auxiliary result.

**Lemma 7 .**

(i) Let (*F*, *A*) be a stable soft set over *X*. Then *x* ∈ (*F*, *A*) if and only if *P*_*a*_^*x*^ ∈ (*F*, *A*).

(ii) (*F*, *A*) is a stable soft set if and only if (*F*^*c*^, *A*) is stable.

**Theorem 8 .**

Every stable soft compact subset (*F*, *A*) of a soft Hausdorff space (*X*, *τ*, *A*) is soft closed.

**Proof**

Suppose that the given condition holds and let *P*_*a*_^*x*^ ∈ (*F*^*c*^, *A*). By hypothesis, (*F*^*c*^, *A*) is stable, we obtain *x* ∈ (*F*^*c*^, *A*). For each *P*_*a*_^*y*_*i*_^ ∈ (*F*, *A*), we obtain *y*_*i*_ ∈ (*F*, *A*). So *x* ≠ *y*_*i*_. Thus there are two disjoint soft open sets (*G*_*i*_, *A*) and (*W*_*i*_, *A*) such that *x* ∈ (*G*_*i*_, *A*) and *y*_*i*_ ∈ (*W*_*i*_, *A*). It follows that {(*W*_*i*_, *A*) : *i* ∈ *I*} forms a soft open cover of (*F*, *A*). Consequently, $\left(F,A)\tilde{\subseteq }{\tilde{⋃}}_{i=1}^{i=n}(W_{i},A\right)$. Putting ${\tilde{⋂}}_{i=1}^{i=n}(G_{i},A)=(H,A)$ and ${\tilde{⋃}}_{i=1}^{i=n}(W_{i},A)=(V,A)$. Now, (*H*, *A*) and (*V*, *A*) are soft open sets such that $(H,A)\tilde{⋂}(V,A)=\tilde{\Phi }$. Therefore $(H,A)\tilde{⋂}(F,A)=\tilde{\Phi }$ and this implies that $\left(H,A)\tilde{\subseteq }(F^{c},A\right)$. Since *P*_*a*_^*x*^ is chosen arbitrary, then (*F*^*c*^, *A*) is soft open. Hence (*F*, *A*) is soft closed.

**Remark 9 .**

It should be noted that [Theorem 34, p.p.5] of [1] is true without imposing a stability of a soft set if we utilize a definition of soft Hausdorff spaces which was introduced in [7].

Finally, we give the following result.

**Theorem 10 .**

Every soft closed subset (*F*, *A*) of a soft Lindelöf space (*X*, *τ*, *A*) is soft Lindelöf.

**Proof**

The proof is similar to that of Theorem 33 of [1].
